# Supplementary material for: Reconfigurable artificial microswimmers with internal feedback
Source: Nat Commun. 2021 Aug 6;12:4762. doi: 10.1038/s41467-021-25108-2 (PMC8346629; doi:10.1038/s41467-021-25108-2)
Supplement: Supplementary file 1 — Supplementary Information [file 41467_2021_25108_MOESM1_ESM.pdf]

SUPPLEMENTARY INFORMATION FOR:

Reconfigurable Artificial Microswimmers with Internal Feedback

L. Alvarez*,[1] M. A. Fernandez-Rodriguez,[1,2] A. Alegria,[3]
S. Arrese-Igor,[3] K. Zhao,[1] M. Kröger,[4] and Lucio Isa*,[5]

[1]*Laboratory for Soft Materials and Interfaces,*
Department of Materials, ETH Zurich, 8093 Zurich, Switzerland

[2]*Biocolloid and Fluid Physics Group,*
Applied Physics Department, Faculty of Sciences,
University of Granada, 18071 Granada, Spain

[3]*Centro de Física de Materiales (CSIC-UPV/EHU),*
Materials Physics Center, 20018 San Sebastián, Spain

[4]*Polymer Physics, Department of Materials,*
ETH Zurich, 8093 Zurich, Switzerland

[5]*Laboratory for Soft Materials and Interfaces,*
Department of Materials, ETH Zurich, 8093 Zurich, Switzerland

Corresponding authors:
* *laura.alvarez-frances@mat.ethz.ch (L.A.),*
* *lucio.isa@mat.ethz.ch (L.I.)*

SUPPLEMENTARY FIGURES

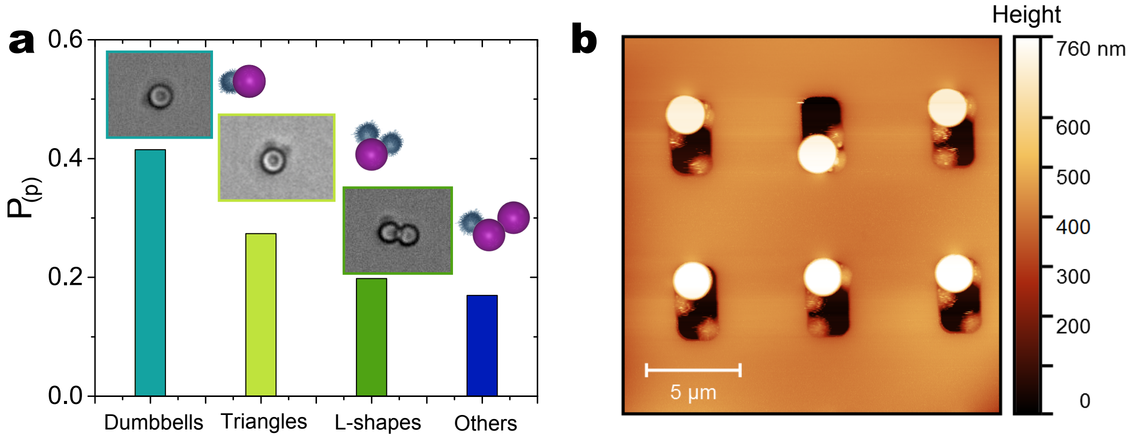

Supplementary Figure 1. **a**, Probability of particle population after sCAPA deposition and transfer to an experimental cell. Insets represent optical microscopy images of the dominant particle populations: dumbbells, triangles and L-shapes with their corresponding schemes. The histogram is obtained by classifying 190 particles after transfer. **b**, AFM image (in water) of the triangular PS-microgel clusters inside the sCAPA template before harvesting, showing some of the assembly defects.

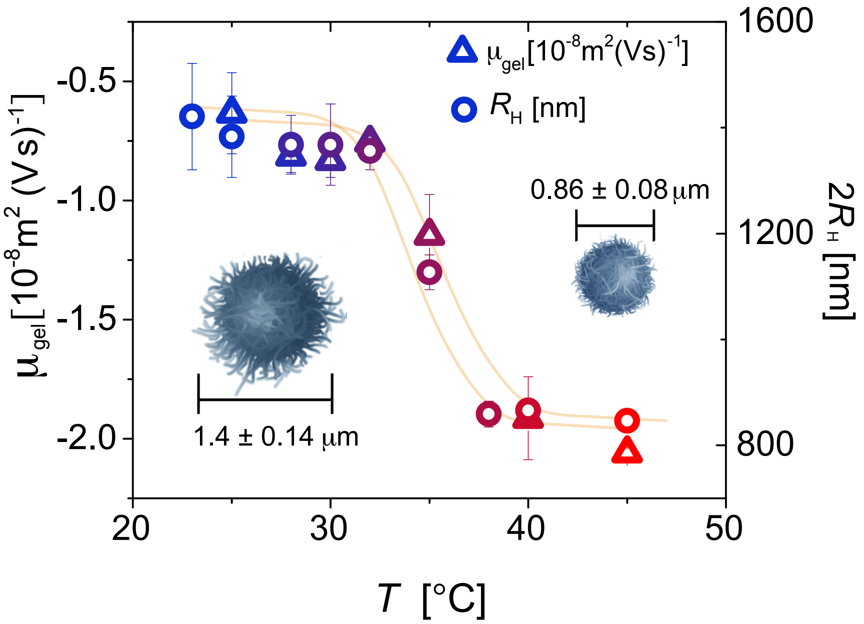

Supplementary Figure 2. Temperature dependence of microgel properties. Electrophoretic mobility (μ_{gel}, open triangles) and hydrodynamic diameter ($2R_{\mathrm{H}}$, open circles) of the colloidal spheres with a given surface charge vs. T. The insets schematically represent the swollen (left) and collapsed (right) microgel before and after its VPTT, respectively. The lines are a guide to the eye.

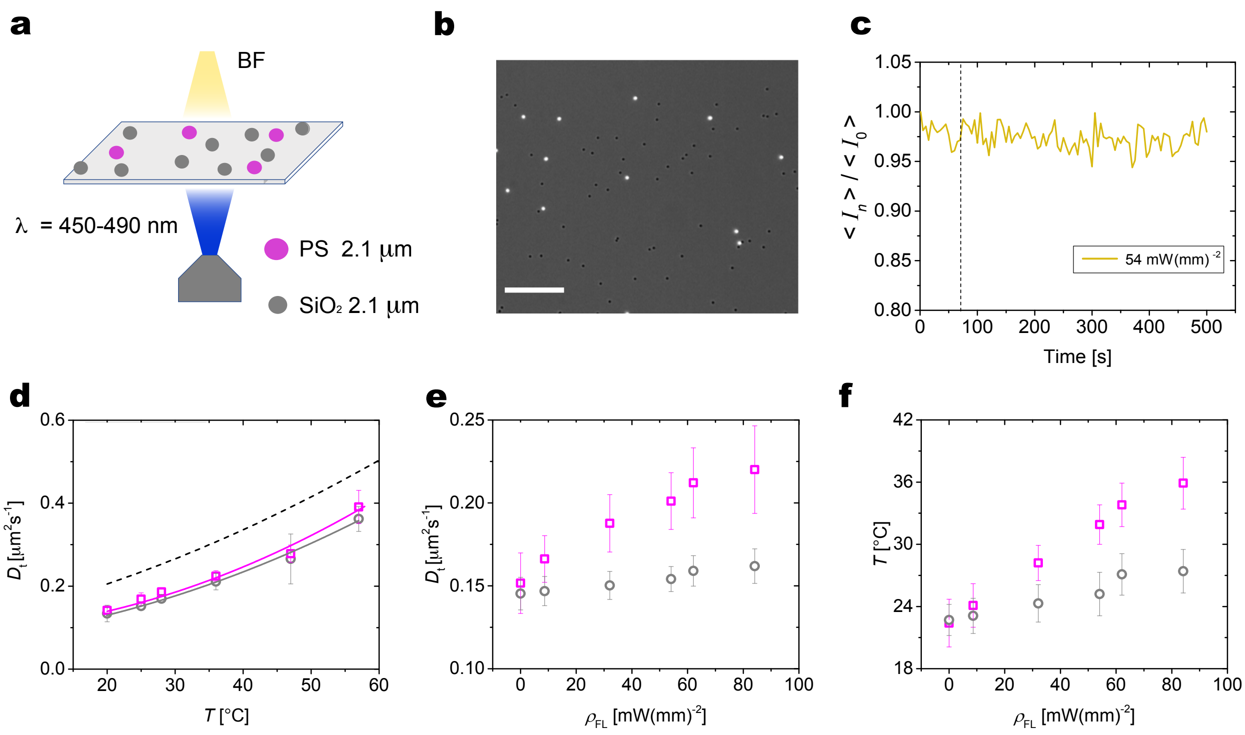

Supplementary Figure 3. **a**, Scheme of the experimental setup mixing epifluorescence (λ = 450–490 nm) and transmission illumination and **b**, resulting microscopy image where PS (bright) and SiO_2 (dark) particles in water can be simultaneously tracked under the same conditions. Scale bar: 20 µm. **c**, Calibration of PS particle fluorescence stability over time at $\rho_{\mathrm{FL}} = 54$ mW(mm)$^{-2}$. The vertical dashed line indicates the maximum time window used in the experiments. **d-f**, Calibration of PS particle heating vs. fluorescence illumination power density. **d**, Translational diffusion coefficient D_{t} for PS (pink) and SiO_2 particles (gray) as a function of T, globally imposed by a Peltier element. The dashed line show the theoretical values for a 2 µm particle in bulk. The solid lines are second order polynomial fits of the data as $D_{\mathrm{t}} = A + BT + CT^2$. **e**, Measured values of D_{t} as a function of ρ_{FL} for both fluorescent PS and non-fluorescent SiO_2 particles. **f**, Conversion between ρ_{FL} and T via the measured values of D_{t} . The extrapolation of T is done from the fitted expression using the diffusivities of both particles under fluorescent illumination from **d**. The data in **f** shows that over the range $\rho_{\mathrm{FL}} < 54$ mW(mm)$^{-2}$, no strong change in diffusivity is seen for the SiO_2 particles. At illumination power densities $\rho_{\mathrm{FL}} > 54$ mW(mm)$^{-2}$ the fluorescent light also induces slight global heating of the sample cell. In all cases the error bars represent the standard deviation of 120 measured particles. The error propagation throughout the extrapolation has been included in the error bars of T.

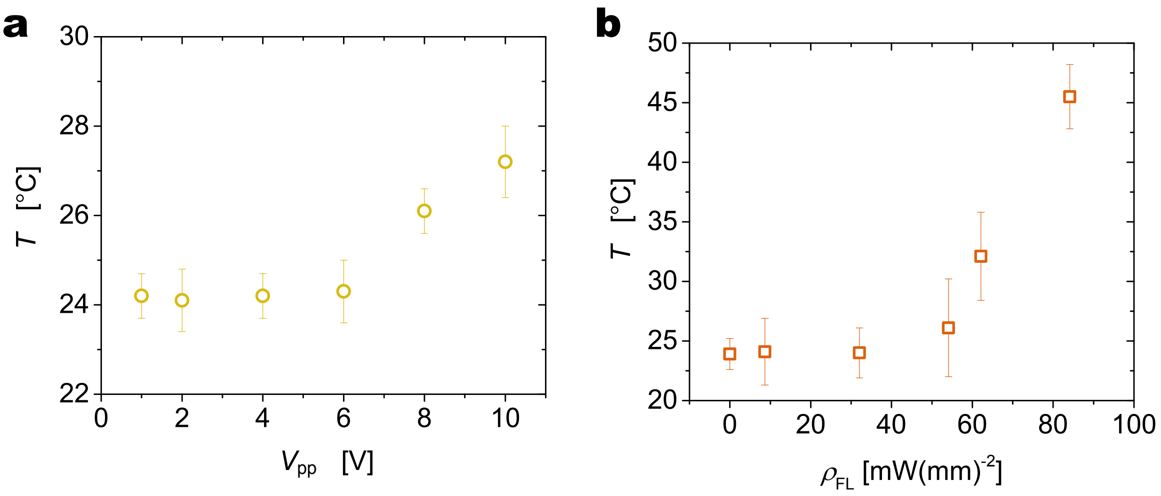

Supplementary Figure 4. Measurement of the global temperature of the sample cell using a thermocouple as a function of **a** applied peak-to-peak voltage different V_{pp} fixing the fluorescence illumination at $\rho_{\mathrm{FL}} = 54$ mW(mm)$^{-2}$, and of **b** the light intensity ρ_{FL} (red) for a fixed $V_{\mathrm{pp}} = 4$ V. The error bars indicate the standard deviation over 11 different measurements.

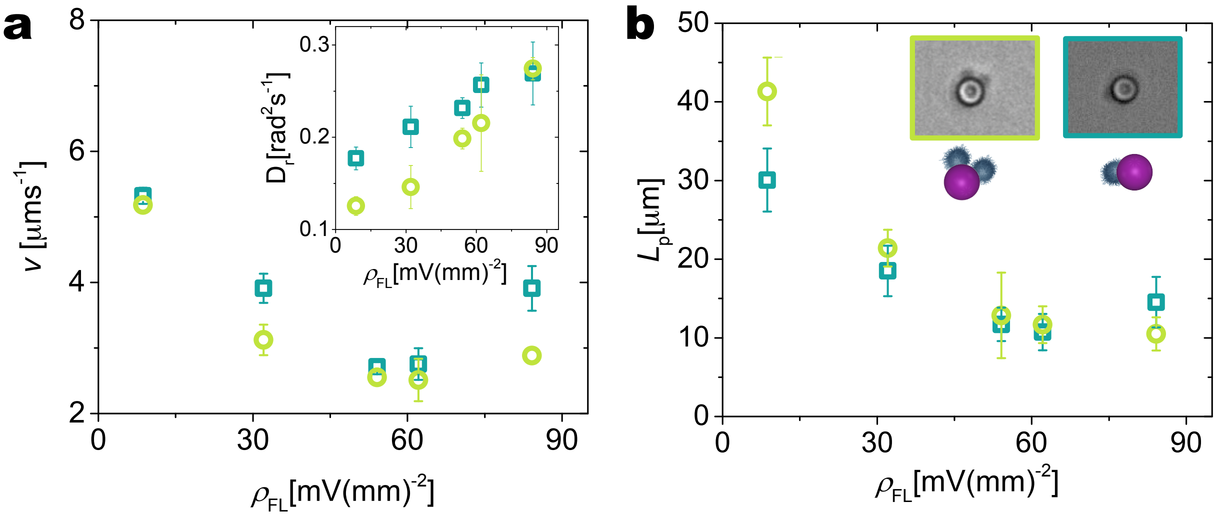

Supplementary Figure 5. **a**, Swimming velocities v and and rotational diffusivity D_{r} of dumbbells (squares) and triangular clusters (circles) as a function of ρ_{FL}. **b**, Persistence length of the trajectories $L_{\mathrm{p}} = v/D_{\mathrm{r}}$ for each cluster shape (same symbols). The inset shows an optical micrograph and the corresponding schematic for each active cluster. The data were obtained over 43 particles for the dumbbells and over 14 particles for the triangles.

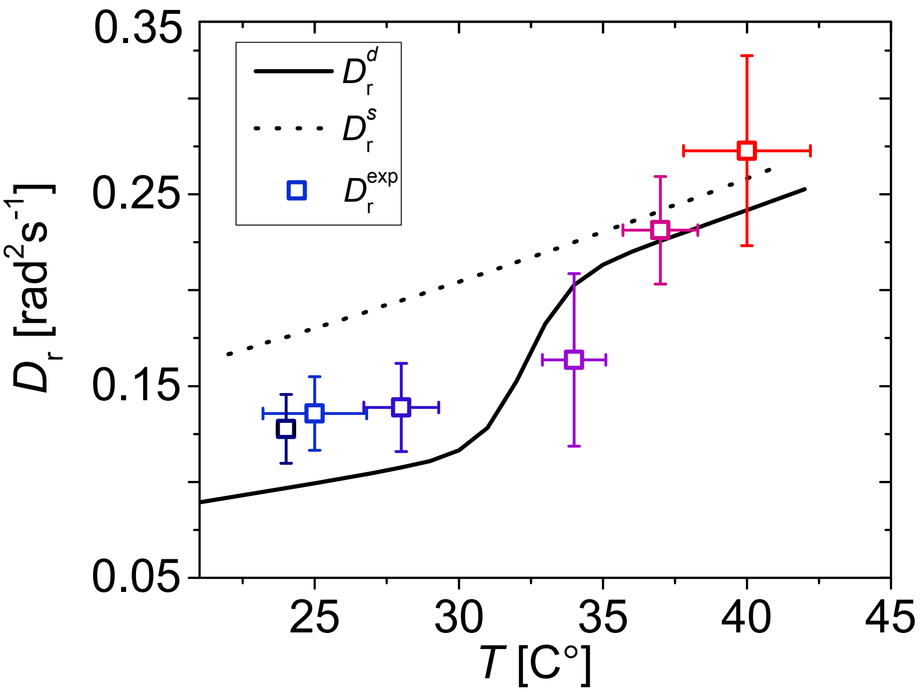

Supplementary Figure 6. Dumbbell's rotational diffusion. Comparison between experimental data (D_r^{exp}, open squares) and theory for a torque-free dumbbell (D_r^d, solid line), and for a sphere (D_r^s, dashed line). Error bars correspond to the standard deviation of D_r^{exp} (y-error bar) and to the uncertainty in measuring T (x-error bar).

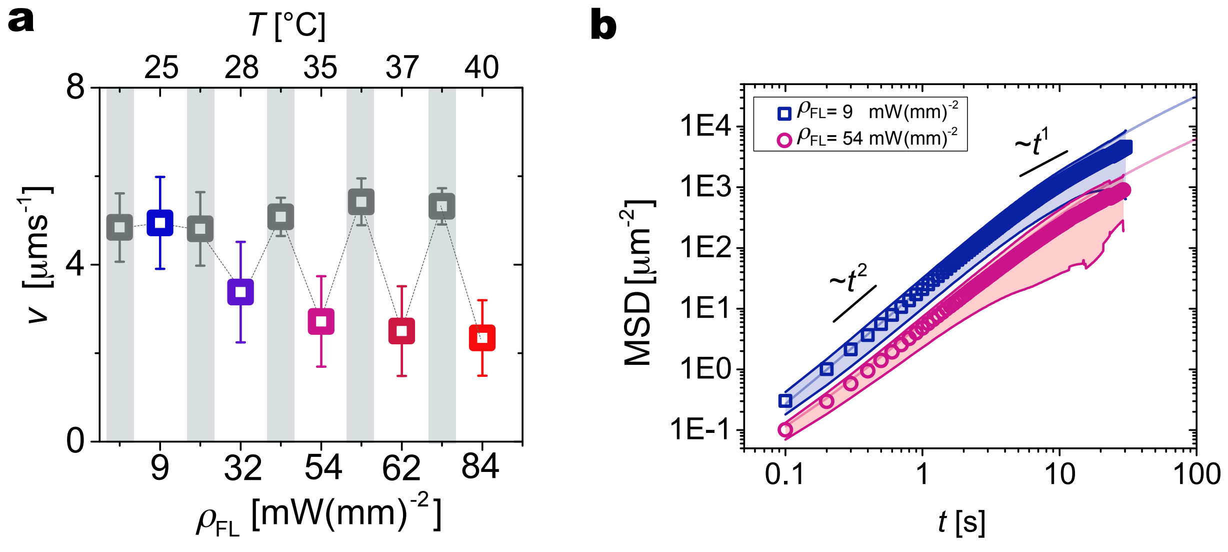

Supplementary Figure 7. Dumbbell's velocity response. **a**, Dumbbell swimming velocity (absolute value) during illumination cycles of no fluorescent light (OFF - gray squares) and different values of ρ_{FL} (red squares) corresponding to different local T (top x-axis). Each data point was collected after a 5-second equilibration. **b**, Mean square displacements of active dumbbells at two values of $\rho_{\mathrm{FL}} = 9$ and 54 mW(mm)$^{-2}$, respectively, below and above the microgel VPTT, showing that the distinctive transition from ballistic $\sim t^2$ to diffusive $\sim t^1$ can be tuned by illumination. Error bars in all cases indicate the standard deviation of values calculated over 150 particles.

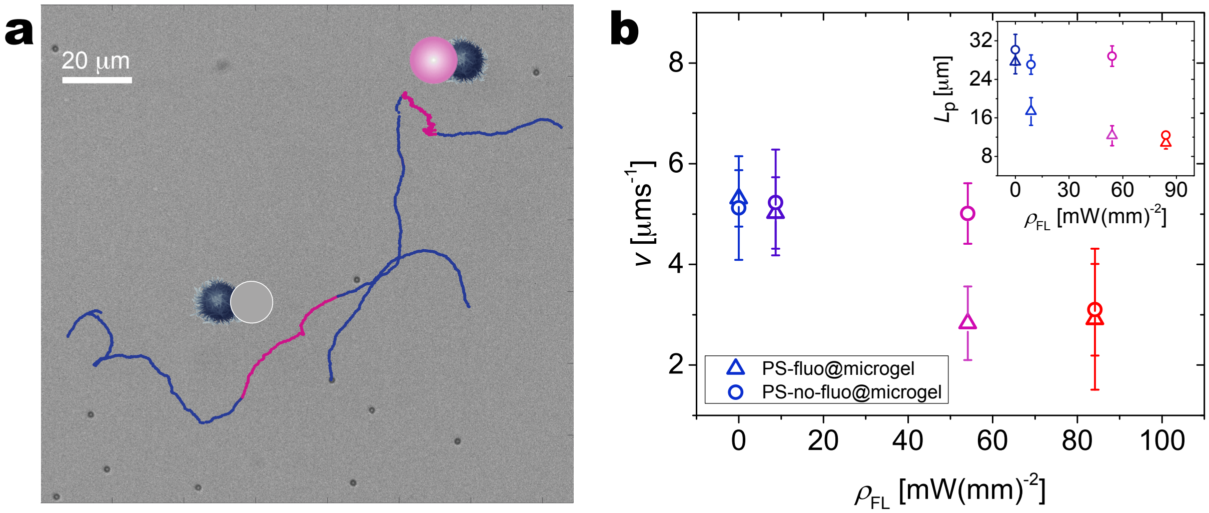

Supplementary Figure 8. **a**, Optical microscopy image and trajectories of dumbbells containing a fluorescent PS particle (pink bead) and a non-fluorescent one (gray), at $\rho_{\mathrm{FL}} = 9$ mW(mm)$^{-2}$ (blue) and $\rho_{\mathrm{FL}} = 54$ mW(mm)$^{-2}$ (magenta) under an homogeneous AC electric field. Scale bar represents 20 μm. **b**, Velocities as a function of ρ_{FL} for the two different types of dumbbells, measured over 56 dumbbells with fluorescent PS and 55 dumbbells with non-fluorescent PS. Inset: Persistence length $L_{\mathrm{p}} = v/D_{\mathrm{r}}$ versus ρ_{FL}. Error bars indicate the standard deviation of the data.

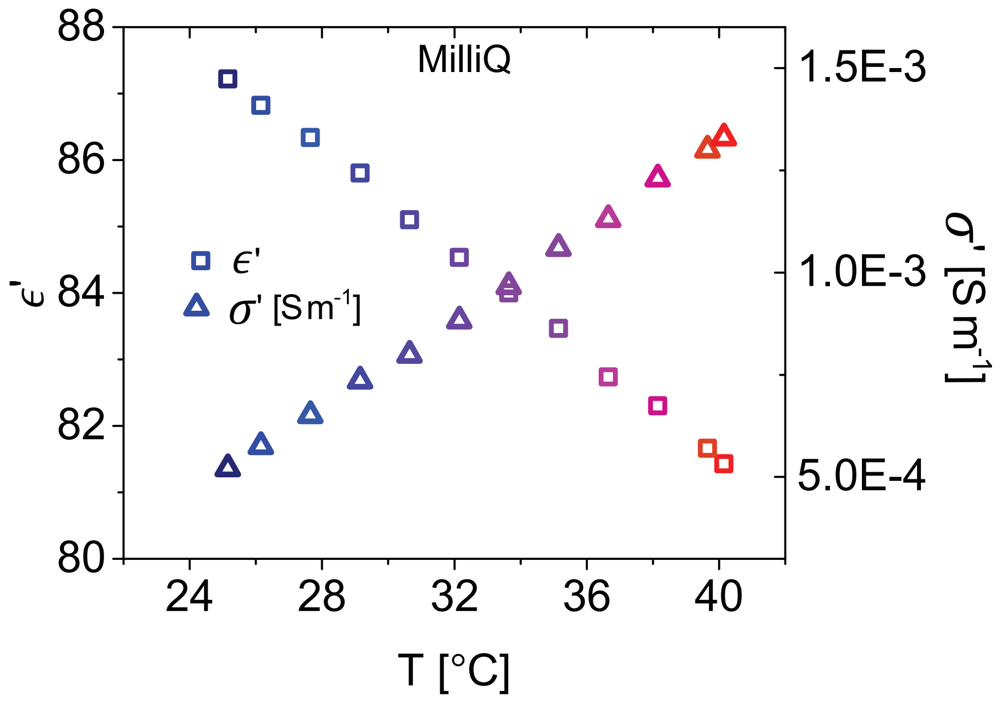

Supplementary Figure 9. $\epsilon'_{\rm m}$ (open squares) and $\sigma'_{\rm m}$ (open triangles) for MilliQ Water as a function of T at a frequency $f = 1$ kHz. The values are obtained by extrapolating the high-frequency plateau to the kHz range.

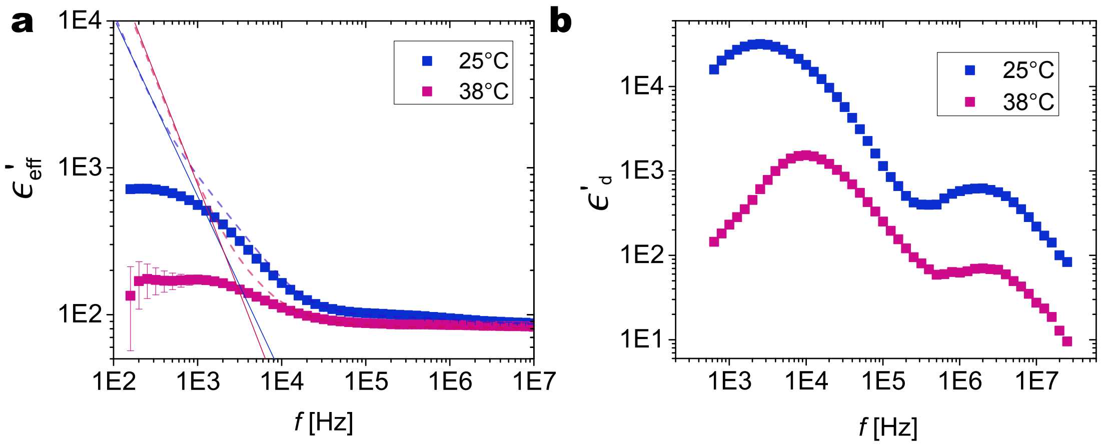

Supplementary Figure 10. Permittivity as a function of frequency for an aqueous suspension of microgels 1% w.t. **a**, Comparison of the raw $\epsilon'_{\rm eff}$ data (dashed lines) with the corrected data (symbols) after subtracting the electrode polarization at 25°C (blue) and 38°C (magenta). The solid lines represent the fits of the electrode polarization effect. **b**, Derivative of the corrected real permittivity of the particle $\epsilon'_{\rm d} = -(2/\pi)\partial\epsilon_{\rm eff}/\partial \ln f$, highlighting the characteristic relaxation events [1].

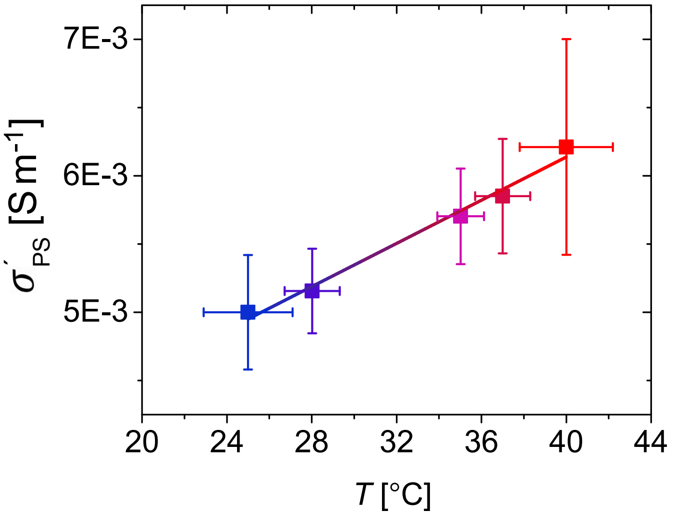

Supplementary Figure 11. Estimated surface conductivity of PS as a function of temperature. Error bars indicate the standard deviation of the temperature values measured in the x-axis, and the error propagation in y-axis from the error of σ'_{m} obtained from the experimental data.

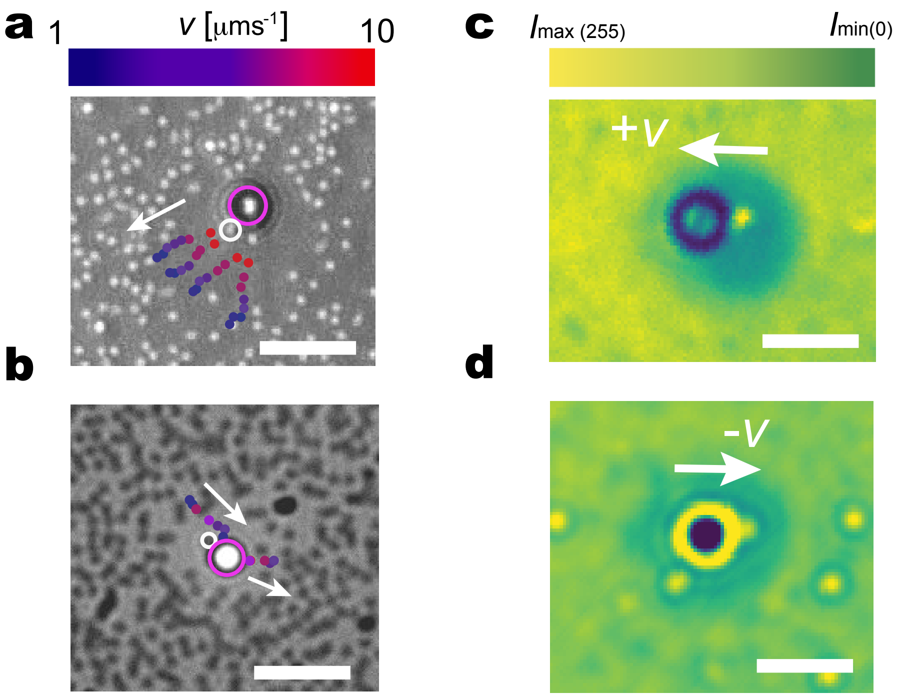

Supplementary Figure 12. Experimental characterization of EHDFs around a dumbbell. **a-b**, Trajectories of PS 700 nm tracer particles around a dumbbell under an external AC electric field of 5 V and 1 kHz at **a**, low [9 mW(mm)$^{-2}$] and **b**, high [54 mW(mm)$^{-2}$] illumination ρ_{FL}. The color coding represents the instantaneous velocity of the tracers. The arrows represent the direction in which the tracers are moving. Scale bars represent 6 μm **c-d**, Integrated intensities of tracers measured over 60 s at low [9 mW(mm)$^{-2}$] **(c)** and high [54 mW(mm)$^{-2}$] **d**, ρ_{FL}. The color coding represents the intensity levels in an 8-bit format from I_{min} (dark green - 0) to I_{max} (bright green - 255). The white arrows indicate the propulsion direction for each condition. Scale bars represent 4 μm.

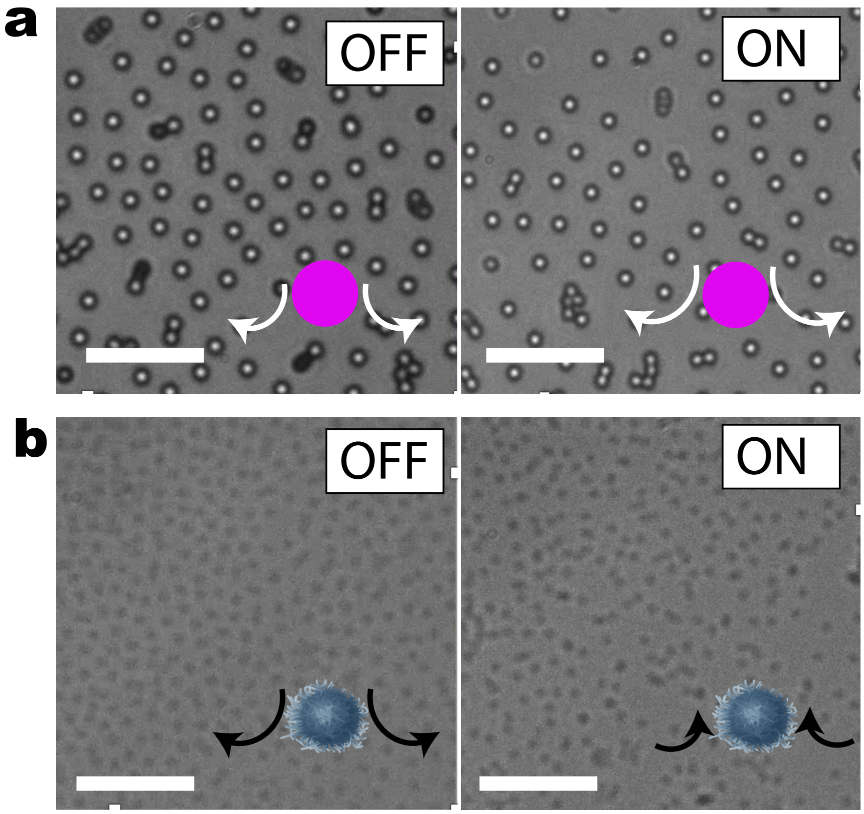

Supplementary Figure 13. Interactions between **a**, PS beads and **b**, microgels, due to EHDFs, under an AC electric ($V_{\mathrm{pp}} = $ 5-6 V) without fluorescence illumination (OFF) and with green light ($\lambda = 500$–550 nm) $\rho_{\mathrm{FL}} = 54$ mW(mm)$^{-2}$ (ON) using a 40× objective. The inset schemes indicate the EHDFs direction and magnitude under the different illumination conditions for each particle type. The scale bars indicate 10 μm.

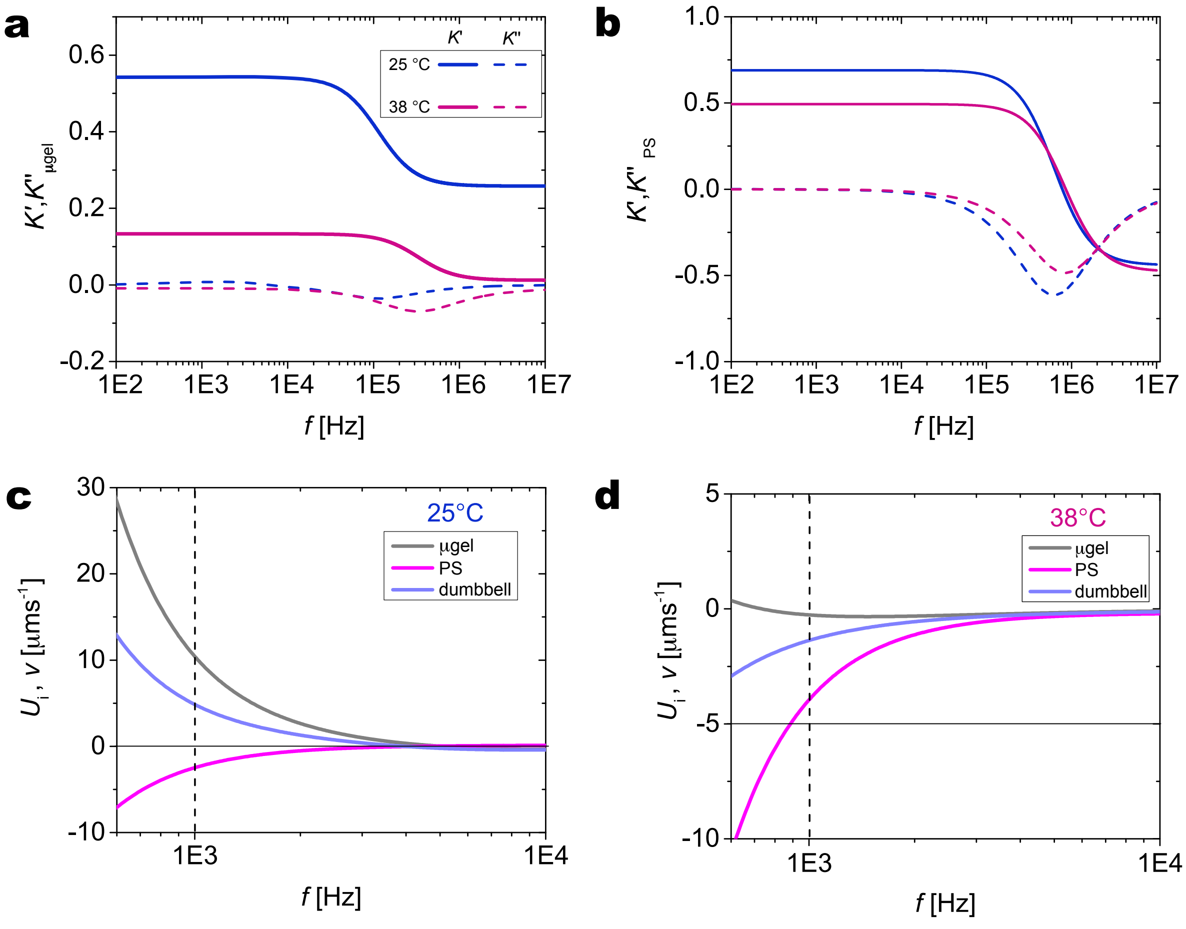

Supplementary Figure 14. Calculation of K' (solid) and K'' (dashed) for **a**, microgels and **b**, PS particles, as a function of frequency at 25°C (blue) and 38°C (magenta). **c-d**, Theoretical prediction of the EHD flow velocities for PS and the microgel, and the resulting dumbbell velocity as a function of frequency, at **c**, 25°C and **d**, 38°C. The dashed vertical line the frequency used in the experiments shown in the main text.

SUPPLEMENTARY NOTES

Supplementary Note 1: Measurement of the dielectric properties of PNIPAM-co-MAA microgels

The dielectric properties ($\epsilon'_{\rm p}$ and $\sigma'_{\rm p}$) of the microgel suspension within a broad range of frequencies were quantified using dielectric spectroscopy. We performed two sets of measurements, one with a microgel suspension of 1 wt% and another one simply using MilliQ water as control measurement. The data were obtained considering Maxwell-Wagner-Sillars' theory, where the complex dielectric function of a heterogeneous mixture of spherical particles relates to the dielectric properties of its components as

$$\frac{\epsilon^*_{\rm eff}-\epsilon^*_{\rm m}}{\epsilon^*_{\rm eff}+2\epsilon^*_{\rm m}}=\frac{\phi\,\epsilon^*_{\rm p}-\epsilon^*_{\rm m}}{\phi\,\epsilon^*_{\rm p}+2\epsilon^*_{\rm m}}=\phi\,K^*, \tag{1}$$

where ϕ is the volume fraction of the particles dispersed in the matrix medium, $\epsilon^*_{\rm eff}$, $\epsilon^*_{\rm p}$ and $\epsilon^*_{\rm m}$ are the complex permittivity of the suspension, of the particle and of the matrix medium, respectively, and K^* is the so called Clausius-Mossotti factor of the particles in the suspension. In particular, within the window of interest for our experiments, the effect of electrode polarization can be fitted by the initial power-law decay of the real part $\epsilon'_{\rm p}$ using the expression af^{-b} [2]. Note that to avoid the EP effect at higher frequencies, we used low-conductivity water ($\sigma < 0.01$ Sm^{-1}). First, we fitted the raw permittivity data and determined the values of a and b. The EP effect was then subtracted from the raw permittivity data at each frequency and temperature. The difference between the raw experimental data and the corrected ones is used Figure 3 of the main manuscript.

Supplementary Note 2: Calculation of the T-dependent propulsion speed for a PS-microgel dumbbell

The theoretical prediction of the EHDFs generated by a single sphere under an applied AC voltage $V_{\rm pp}e^{-j\omega t}$ has been studied and derived by N. Wu *et al.* in previous works [3, 4] (the expression has been corrected from the original article, information shared with the authors via private communication). The EHDFs depend on D (the diffusion coefficient of the ions in the liquid), κ^{-1} (the Debye length) and H (the half-separation between the electrodes, 60 µm in our case). Therefore, the velocity of the EHDF $U_{\rm i}$ around a single particle of radius $R_{\rm i}$ at a given reference distance $r_{\rm i}$ at which the EHD is evaluated, within 2 electrodes separated by a distance $2H$, can be calculated using

$$U_{\rm i}=\beta\frac{C}{\eta}\frac{K'+K''\bar{\omega}}{1+\bar{\omega}^2}\frac{3(r_{\rm i}/R_{\rm i})}{2\left[1+(r_{\rm i}/R_{\rm i})^2\right]^{5/2}},\qquad C=\epsilon_{\rm m}\epsilon_0 H\left(\frac{V_{\rm pp}}{2H}\right)^2 \tag{2}$$

where $\bar{\omega} = \omega H/\kappa D$ and η is the fluid viscosity. To evaluate the EHD for each single lobe, we consider r_i as the distance from the center of the evaluated lobe to the center of the adjacent particle ($r = R_{\mathrm{\mu gel}} + R_{\mathrm{PS}}$). Therefore, as the radius of the microgel $R_{\mathrm{\mu gel}}$ varies with temperature, r_i also changes. Finally, K' and K'' are the real and imaginary part of the Clausius-Mosotti factor $K^* = K' + jK''$ [5]. They play a key role in calculating U_i, as they determine the sign of the EHDF. The Clausius-Mossotti factor is often used to describe the polarizability of a particle suspended in a fluid. It dictates magnitude and sign of the induced dipole moment, and therefore also the distortion of the fluid flows of charged particle under an AC electric field [5, 6]. In particular:

$$K' = \frac{\omega^2 \epsilon_0^2 (\epsilon'_\mathrm{p} - \epsilon'_\mathrm{m})(\epsilon'_\mathrm{p} + 2\epsilon'_\mathrm{m}) + (\sigma'_\mathrm{p} - \sigma'_\mathrm{m})(\sigma'_\mathrm{p} + 2\sigma'_\mathrm{m})}{\omega^2 \epsilon_0^2 (\epsilon'_\mathrm{p} + 2\epsilon'_\mathrm{m})^2 + 2(\sigma'_\mathrm{p} + 2\sigma'_\mathrm{m})^2} \tag{3}$$

$$K'' = \frac{\omega \epsilon_0 (\epsilon'_\mathrm{p} - \epsilon'_\mathrm{m})(\sigma'_\mathrm{p} + 2\sigma'_\mathrm{m}) - \epsilon_0 (\epsilon'_\mathrm{p} + 2\epsilon'_\mathrm{m})(\sigma'_\mathrm{p} - \sigma'_\mathrm{m})}{\omega^2 \epsilon_0^2 (\epsilon'_\mathrm{p} + 2\epsilon'_\mathrm{m})^2 + 2(\sigma'_\mathrm{p} + 2\sigma'_\mathrm{m})^2} \tag{4}$$

Here, ϵ_p and ϵ_m are the permittivities of the particle and the medium, respectively, and σ'_p and σ'_m are the conductivities (Sm^{-1}) of the particle and the medium, respectively. For the calculation of K' and K'' of the microgels, we use the corrected experimental values of ϵ'_{eff} and σ'_{eff} and the volume fraction of the solution at each temperature as shown in Supplementary Eq. (1), interpolating for the range of frequencies and temperatures studied here. The values of ϵ'_m and σ'_m are extrapolated from the measured values of their high-frequency plateaus. For the PS particles, we consider $\epsilon'_\mathrm{p} = 2.5$ as a constant value for the range of experimental temperatures and frequencies [7]. The estimation of σ'_p for PS was done considering that [5]:

$$\sigma'_\mathrm{p} = \sigma_\mathrm{b} + \frac{2K_\mathrm{s}}{r} \tag{5}$$

where σ'_b is the bulk conductivity (in Sm^{-1}), where for dielectric particles $\sigma_\mathrm{b} \approx 0$ [8], [9], and K_s the surface conductance (in S) of the particle. K_s can be further split into the Stern layer conductance K_{sl} and the diffuse layer conductance K_d

$$K_\mathrm{s} = K_{\mathrm{sl}} + K_\mathrm{d} \tag{6}$$

where K_{sl} is known for polystyrene latex in similar experimental conditions, i.e. $K_{\mathrm{sl}} \approx 0.5$ nS [3, 10]. The value of K_d can be calculated as

$$K_\mathrm{d} = \frac{2\sigma_\mathrm{m}}{\kappa} \left[\frac{D^+}{D^+ + D^-} \left(e^{-\frac{z\zeta_\mathrm{p} e}{2k_\mathrm{B}T}} - 1 \right) (1 + 3m^+) + \frac{D^-}{D^+ + D^-} \left(e^{+\frac{z\zeta_\mathrm{p} e}{2k_\mathrm{B}T}} - 1 \right) (1 + 3m^-) \right] \tag{7}$$

where σ'_m is the medium conductivity, e the electron charge, z the valence of the ions, and ζ_p is the zeta-potential of the particle ($\zeta_\mathrm{PS} = -71$ mV) and $D^\pm$ the diffusion coefficients of the negatively and positively charged ions. In our experiments, we primarily use MilliQ water, but also carry out control experiments in 10^{-5} M of KCl, so we consider the ionic species K^+ and Cl^-. The parameters $m^\pm$ are given as [11]

$$m^\pm = \frac{2\epsilon_\mathrm{m}\epsilon_0}{3\eta D^\pm}\left(\frac{k_\mathrm{B}T}{ze}\right)^2 \tag{8}$$

which describe the contribution of electro-osmotic ion flux to K_d, where η is the viscosity of the fluid. We therefore estimate the magnitude and behavior of σ'_p for the PS particles (Supplementary Figure 11) as a function of temperature, considering the temperature dependence of the diffusion coefficient of K^+ and Cl^- at 10^{-5} M [12]. At this salt concentration, the propulsion behavior of the dumbbells is the same as in the experiments with only MilliQ water ($\sigma'_\mathrm{m} \approx 5 \times 10^{-3}$ Sm^{-1}).

Supplementary Note 3: Experimental observation of EHDFs

The direction of the EHDFs can be experimentally observed by using small fluorescent tracers. The tracers at each side of a dumbbell might be ejected (repulsive) or taken in (attractive), revealing the direction of the EHDF. At low light intensities, we observe that tracers are strongly ejected away from the microgel, while the same phenomenon occurs with a lower intensity in the proximity of the PS lobe. This indicates that, albeit both flows are repulsive, they are highly asymmetric and the overall behavior is dominated by the flow around the microgel surface, causing the dumbbell to propel with the PS lobe in front. When the microgel shrinks at higher light intensities, the sign of its EHD flow changes, and the magnitude of the flow reduces. At the same time, the sign of the PS EHD remains the same but increases slightly due to local viscosity decrease. The combination of the latter effects causes an inversion of the propulsion direction of the dumbbell, swimming with the microgel in front.

SUPPLEMENTARY REFERENCES

[1] Jiménez, M., Arroyo, F., van Turnhout, J. & Delgado, A. Analysis of the dielectric permittivity of suspensions by means of the logarithmic derivative of its real part. *J. Colloid Interf. Sci.* **249**, 327–335 (2002).

[2] Su, W., Zhao, K., Wei, J. & Ngai, T. Dielectric relaxations of poly(N-isopropylacrylamide) microgels near the volume phase transition temperature: Impact of cross-linking density distribution on the volume phase transition. *Soft Matter* **10**, 8711–8723 (2014).

[3] Ma, F., Yang, X., Zhao, H. & Wu, N. Inducing propulsion of colloidal dimers by breaking the symmetry in electrohydrodynamic flow. *Phys. Rev. Lett.* **115**, 208302 (2015).

[4] Ma, F., Wang, S., Wu, D. T. & Wu, N. Electric-field-induced assembly and propulsion of chiral colloidal clusters. *Proc. Natl. Acad. Sci. USA* **112**, 6307–6312 (2015).

[5] Pethig, R. *The Clausius–Mossotti Factor*, chap. 6, 119–144 (John Wiley & Sons, Hoboken, NJ, 2017).

[6] Shilov, V. *et al.* Polarization of the electrical double layer. time evolution after application of an electric field. *J. Colloid Interf. Sci.* **232**, 141–148 (2000).

[7] Ellingson, S. *Electromagnetics* (Virginia Tech Publishing, Blacksburg, VA, 2018).

[8] O'Konski, C. T. Electric properties of macromolecules. V. theory of ionic polarization in polyelectrolytes. *J. Phys. Chem.* **64**, 605–619 (1960).

[9] Ermolina, I. & Morgan, H. The electrokinetic properties of latex particles: Comparison of electrophoresis and dielectrophoresis. *J. Colloid Interf. Sci.* **285**, 419–28 (2005).

[10] Ristenpart, W. D., Aksay, I. A. & Saville, D. A. Electrohydrodynamic flow around a colloidal particle near an electrode with an oscillating potential. *J. Fluid Mech.* **575**, 83–109 (2007).

[11] Yang, X., Johnson, S. & Wu, N. The impact of stern-layer conductivity on the electrohydrodynamic flow around colloidal motors under an alternating current electric field. *Adv. Intell. Syst.* **1**, 1900096 (2019).

[12] Harned, H. S. & Nuttall, R. L. The Differential Diffusion Coefficient of Potassium Chloride in Aqueous Solutions. *J. Amer. Chem. Soc.* **71**, 1460–1463 (1949).
