# Supplementary material for: Reconfigurable artificial microswimmers with internal feedback
Source: Nat Commun. 2021 Aug 6;12:4762. doi: 10.1038/s41467-021-25108-2 (PMC8346629; doi:10.1038/s41467-021-25108-2)
Supplement: Supplementary file 2 — Description of Additional Supplementary Files [file 41467_2021_25108_MOESM2_ESM.pdf]

Description of Additional Supplementary Files

File name: Supplementary Movie 1
Description: Reconfiguration of a PS-microgel assembly upon local temperature change via fluorescence illumination cycles (ON and OFF).

File name: Supplementary Movies 2 and 3
Description: Dynamical control of active PS-microgel assemblies under AC electric field (f= 1kHz and Vpp= 4V) during various ON and
OFF cycles of fluorescent illumination. Only the fluorescent PS particles are visible (40×magnification in different ROIs).

File name: Supplementary Movie 4
Description: Control experiments using fluorescent and non-fluorescent PS to fabricate the dumbbells, switching the fluorescent illumination
on and off.

File name: Supplementary Movie 5
Description: Reversal of swimming direction of an active PS-microgel dumbbell due to an increase of local temperature when illuminating at
high fluorescence intensity (63x magnification).

File name: Supplementary Movies 6 and 7.
Description: Active motion of L-shaped clusters at different illumination conditions exhibiting chirality inversion upon selfreconfiguration. The Movie S6 is recorded with the fluorescence channel only and Movie S7 by combining the transmission channel with fluorescence.

File name: Supplementary Movies 8 and 9
Description: Interactions mediated by EHDFs for PS particles and microgels at a fixed AC field of Vpp= 5V undergoing a global change of temperature mediated by illumination using green light (band-pass filter λ= 500–550nm) with ρFL= 54mW(mm)−2. Light-absorption by the gold film coating the electrodes causes local heating above the VPTT of the microgels. Upon heating, the flow surrounding the PS particles remains repulsive, while it goes from repulsive to attractive for the microgels, as shown by the melting of a particle lattice into local transient clusters.
